# Bacterial Microbiome Differences between the Roots of Diseased and Healthy Chinese Hickory (*Carya cathayensis*) Trees

**DOI:** 10.4014/jmb.2304.04054

**Published:** 2023-07-21

**Authors:** Xiao-Hui Bai, Qi Yao, Genshan Li, Guan-Xiu Guan, Yan Fan, Xiufeng Cao, Hong-Guang Ma, Mei-Man Zhang, Lishan Fang, Aijuan Hong, Dacai Zhai

**Affiliations:** 1 College of Life and Environment Science, Huangshan University, Huangshan, Anhui 245041, P.R. China; 2 Forestry Science and Technology Promotion Center of Shexian, Huangshan, Anhui 245200, P.R. China; 3 School of Life Science and Technology, Henan Institute of Science and Technology, Xinxiang, Henan 453003, P.R. China; 4 Huangshan Tianzhiyuan Agricultural Products Co., Ltd., Huangshan, Anhui 245213, P.R. China; 5 Huangshan Shanye Local Specialty Co., Ltd., Huangshan, Anhui 245200, P.R. China

**Keywords:** *Carya cathayensis*, root tissue, rhizosphere soil, bulk soil, bacterial communities

## Abstract

*Carya cathayensis* is an important economic nut tree that is endemic to eastern China. As such, outbreaks of root rot disease in *C. cathayensis* result in reduced yields and serious economic losses. Moreover, while soil bacterial communities play a crucial role in plant health and are associated with plant disease outbreaks, their diversity and composition in *C. cathayensis* are not clearly understood. In this study, Proteobacteria, Acidobacteria, and Actinobacteria were found to be the most dominant bacterial communities (accounting for approximately 80.32% of the total) in the root tissue, rhizosphere soil, and bulk soil of healthy *C. cathayensis* specimens. Further analysis revealed the abundance of genera belonging to Proteobacteria, namely, *Acidibacter*, *Bradyrhizobium*, *Paraburkholderia*, *Sphaerotilus*, and *Steroidobacter*, was higher in the root tissues of healthy *C. cathayensis* specimens than in those of diseased and dead trees. In addition, the abundance of four genera belonging to Actinobacteria, namely, *Actinoallomurus*, *Actinomadura*, *Actinocrinis*, and *Gaiella*, was significantly higher in the root tissues of healthy *C. cathayensis* specimens than in those of diseased and dead trees. Altogether, these results suggest that disruption in the balance of these bacterial communities may be associated with the development of root rot in *C. cathayensis*, and further, our study provides theoretical guidance for the isolation and control of pathogens and diseases related to this important tree species.

## Introduction

Soil microorganisms, including bacteria, fungi, viruses, algae, and other microbial groups, are considered the most important and active components of the soil. They can change the physical and chemical characteristics of the soil through their biological activities and play an important role in maintaining soil ecological balance and promoting plant growth [1]. Therefore, most ecological niches contain numerous microorganisms [2].

Plants can produce different nutrients that help attract microbial colonization, and therefore, microorganisms are found in the roots, stems, leaves, and fruits of plants. Some microorganisms benefit plant growth and reproduction, whereas some can induce diseases in plants or lead to their death. The rhizosphere, known as the second genome of plants [3], is essential in maintaining the normal functioning of the soil ecosystem. Rhizosphere soils are strongly influenced by the root system and surrounding environment, which together host strong interactions among roots, soil, and microorganisms [4]. For instance, approximately 40% of the carbon fixed through photosynthesis is released through root secretions, of which, 11% is retained in rhizosphere sedimentation [5, 6]. Microorganisms found in the rhizosphere are vital in regulating biotic and abiotic stress tolerance and controlling pests and diseases [7]. It is therefore necessary to understand the relationship between rhizosphere microbiota and plants.

Chinese hickory (*Carya cathayensis*), an important economic nut tree, is narrowly endemic to eastern China in the wild [8]. The species is naturally distributed in moist valleys at altitudes of 500–1,200 m in China’s Zhejiang and Anhui provinces [9]. Owing to its high nutritional and economic value, *C. cathayensis* is widely cultivated in Anhui Province and is grown in a purely forested mode [10]. However, with the large-scale cultivation of *C. cathayensis* and extensive use of herbicides in recent years, the incidence of root rot of unknown causes has increased, resulting in great economic losses to local fruit farmers.

Previous studies have revealed that the composition, function, and diversity of soil bacterial communities have a major effect on plant health and are associated with plant disease outbreaks [11-13]. However, our understanding of the diversity and composition of bacterial communities in *C. cathayensis* remains elusive. In this study, we analyzed changes in the bacterial community structure in the root tissue, rhizosphere soil, and bulk soil of healthy *C. cathayensis* specimens using high-throughput sequencing technology. Altogether, the findings reveal differences in the bacterial communities among specimens of *C. cathayensis* in different states of health during the occurrence of root rot and provide theoretical guidance for the isolation of related pathogens and disease control.

## Materials and Methods

### Plant Classification and Experimental Design

*C. cathayensis* trees with a height of 7.5–8.5 m and planted at a distance of 30–50 m were randomly selected as described in a previous study [15]. To analyze differences in bacterial communities between diseased and healthy examples of *C. cathayensis*, trees were divided into three groups as follows: healthy plants (NP group), diseased plants (SP group), and dead plants (DP group). In addition, samples collected from each group were divided into root tissue (RT), rhizosphere soil (RS) [14], and bulk soil (BS) groups to compare bacterial communities more accurately. All samples were used for DNA extraction to amplify and sequence the bacterial *16S rRNA* gene. Approximately 3–6 individual trees were randomly selected from each group for analysis.

### Soil Sample Collection and Processing

Test soil was collected from *C. cathayensis* plantations (30°02'N, 118°48'E, 321 m asl) in Cikeng, Shexian County, Anhui Province, China. After removing 3–5 cm of surface soil, BS and RS samples were collected from a depth of 4–20 cm at each tree. RS samples were collected according to a previously reported method [16]. BS samples were collected from three randomly selected areas at a distance of approximately 50 cm from the trees and were stored in sterile aluminum boxes [17]. Root tissues were collected from each tree and mixed as a sample. All samples had more than three replicates. The samples were labeled and packaged with dry ice for transportation.

The root tissues were washed under tap water and sterile water for 5 min as described previously [18]. The tissues were cut into small pieces and homogenized with 1 ml of phosphate-buffered saline (PBS) (pH, 7.4) containing 10 mM Na_2_HPO_4_, 1.8 mM KH_2_PO_4_, 2.7 mM KCl, and 137 mM NaCl using a mortar and pestle. After centrifugation at 8,000 ×*g* for 15 min, precipitates containing endophytic samples were collected and stored at -80°C until DNA extraction.

### Extraction of Genomic DNA

Genomic DNA was extracted from 0.5 g of each sample using the CTAB method [19]. The concentration and purity of DNA were determined via agarose gel electrophoresis (1% gels). According to the concentration, the DNA was diluted to a concentration of 1 ng/μl with sterile water.

### Quantitative and Qualitative Analyses of PCR Products

The V4 region of 16S rDNA in each sample was amplified using a specific primer pair (341F: 5'-CCTAYGGGR BGCASCAG-3', 806R: 5'-GGACTACNNGGGTATCTAAT-3') with the barcode [20]. PCR was performed using Phusion High-Fidelity PCR Master Mix (New England Biolabs, USA), and PCR products were detected via agarose gel electrophoresis (2% gels). Samples with a bright main strip between 400 and 450 bp were selected for further experiments.

### Library Preparation and Sequencing

Sequencing libraries were generated using the TruSeq DNA PCR-Free Sample Preparation Kit (Illumina, USA) according to the manufacturer’s instructions. The quality of the libraries was assessed on the Qubit@ 2.0 Fluorometer (Thermo Fisher Scientific, USA) and the Agilent Bioanalyzer 2100 system (Agilent, USA). The libraries were sequenced on the Illumina HiSeq2500 platform, resulting in the generation of 250-bp paired-end reads. The paired-end reads were assigned to samples based on their unique barcode and truncated by cutting off the barcode and primer sequence. Subsequently, the reads were merged using the FLASH (V1.2.7) tool [21].

### Processing of Sequencing Data

Raw data were processed as described previously [22] using default settings. Briefly, raw tags were loaded to the EasyAmplicon pipeline for quality control and filtering. Clean data were clustered into operational taxonomic units (OTUs) at 97% similarity or denoised into amplicon sequence variants (ASVs). The phylogenetic affiliation of each *16S rRNA* gene sequence was analyzed using the EasyAmplicon pipeline based on the Ribosomal Database Project version 18 [23]. Richness and diversity indices including Chao1, ACE, Richness, Shannon, and Simpson were evaluated using the EasyAmplicon pipeline [22]. Feature tables and phylogenetic trees were generated using the EasyAmplicon pipeline to calculate all types of α- and β-diversity metrics.

### Statistical Analysis

Statistical analysis was performed using the R software (version 4.2.2). The statistical significance of the α-diversity between DP, NP, and SP groups was evaluated using ANOVA with Tukey HSD, and a *p*-value of < 0.05 was considered statistically significant. The permutational multivariate analysis of variance (PERMANOVA) by Adonis was used for β-diversity analysis. We used the edgeR package [24] to calculate the differences in ASV abundance between DP, NP, or SP groups, with the Benjamini–Hochberg method to control the false discovery rate.

### Data Availability

The clean sequence data have been deposited in the National Genomics Data Center [25] and can be obtained with the accession number CRA009151 at https://ngdc.cncb.ac.cn/gsa.

## Results

### Disease Symptoms in *C. cathayensis*

**Fig. 1:**
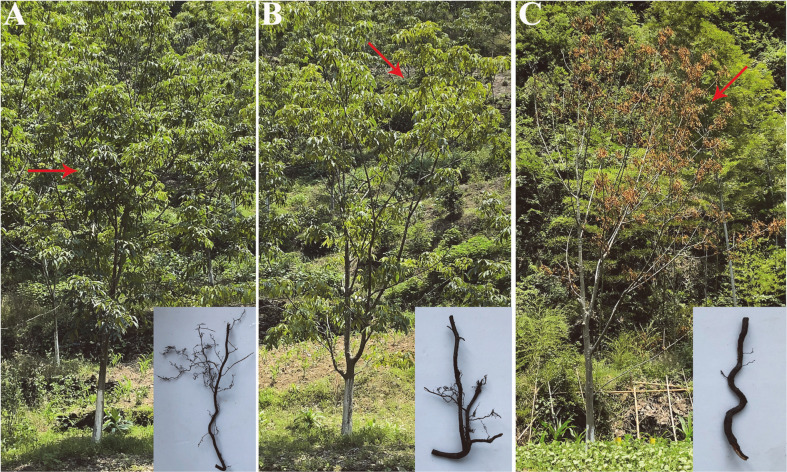
Images depicting healthy (**A**) diseased (**B**) and dead (**C**) *C. cathayensis* specimens. (**A, B**, and **C** insets) For observing the characteristics of root tissue, close-up views of the root tissue of each group are shown.

Chinese hickory (*C. cathayensis*) trees are the main source of income for fruit farmers in Sanyang, Shexian County, where chemical fertilizers and herbicides are used in large quantities to increase yield. In recent years, we have found some *C. cathayensis* specimens that turned yellow and wilted (referred to as diseased plants) and died suddenly (referred to as dead plants). Careful observation of the above-ground parts of *C. cathayensis* showed that the leaves of healthy trees were dark green and naturally extended (Fig. 1A). However, the leaves of diseased trees were yellow and pendulous. The leaves of diseased trees were able to spread naturally on rainy days without wilting; however, once the rain stopped and the sky cleared, the leaves would exhibit wilting symptoms after 2 days (Fig. 1B). Meanwhile, the leaves of dead *C. cathayensis* specimens appeared yellow (Fig. 1C). No other abnormalities were observed in the above-ground part of healthy, diseased, and dead trees.

On digging out the roots of *C. cathayensis* specimens, we found that healthy trees had developed roots with many lateral and fibrous roots. In particular, numerous white fibrous roots were visible (Fig. 1A). The roots of diseased trees were decomposed, and the abundance of lateral and fibrous roots was lower than that in healthy trees. Moreover, lateral or fibrous roots fell off a lot when the roots were washed with water. The root phloem was found separated from the xylem, and the xylem turned brown or black (Fig. 1B). Dead *C. cathayensis* specimens had fewer lateral and fibrous roots, and their phloem and xylem were detached, with the xylem turning black (Fig. 1C).

Root rot is the most important plant disease worldwide, and typical symptoms include browning, softening, decay, and eventual death of roots. Plant leaves turn yellow and wilt, have retarded elongation, and may die [26, 27]. Overall, the symptoms exhibited by *C. cathayensis* specimens in this study were consistent with the typical symptoms of root rot disease. Given that bacteria, viruses, oomycetes, and fungi can cause root rot [26, 27], we analyzed the root microbiota of *C. cathayensis* specimens using amplicon sequencing technology to provide data for subsequent identification of the causative agent.

### Qualitative Analysis of Sequencing Data

**Fig. 2:**
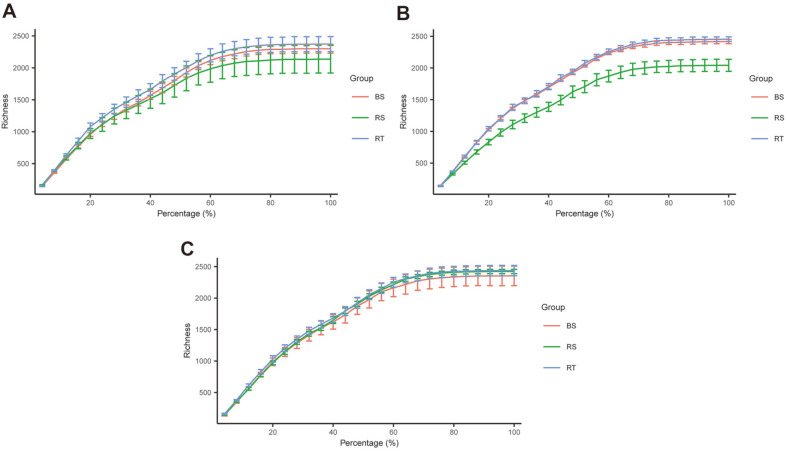
Rarefaction curves of different groups of *C. cathayensis*. Sample taxonomic richness increases with increasing sequencing depth. Each error bar represents standard error. (**A**) healthy plant (NP); (**B**) diseased plant (SP); (**C**) dead plant (DP).

A total of 2,298,108 sequence reads of *16S rRNA* gene amplicons were obtained from 27 samples. After quality control, a total of 955,374 clean reads of *16S rRNA* gene amplicons were obtained (ranging from 20,072 to 39,624), producing an average of 4,661 amplicon sequence variants (ASVs) with 97% identity. To verify the accuracy of results, rarefaction curves were plotted to analyze the relationship between species richness and sequencing depth. The curves tended to be saturated with an increase in sequencing depth in each group, indicating that the sequencing depth was adequate for covering the entire bacterial microbiome (Fig. 2).

### Alpha Diversity

**Fig. 3:**
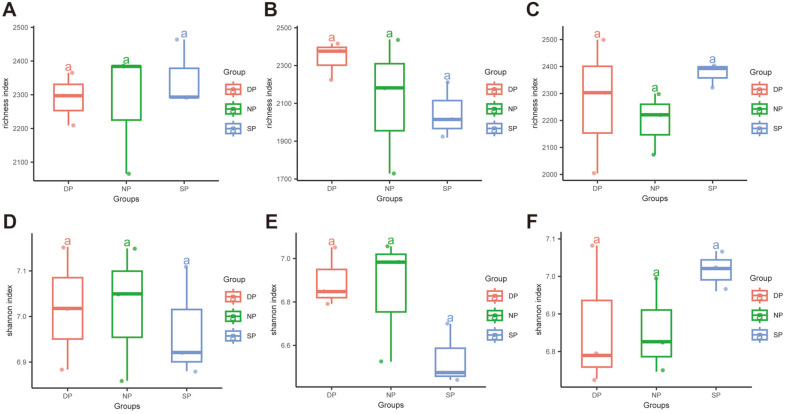
Boxplots of the Richness and Shannon indexes in the samples of RT, RS, and BS. The Richness index of (**A**) RT, (**B**) RS, and (**C**) BS. The Shannon index of (**D**) RT, (**E**) RS, and (**F**) BS. RT, root tissue; RS, rhizosphere soil; BS, bulk soil. NP, healthy plant; SP, diseased plant; DP, dead plant. Box plots show the first (25%) quartile, the third (75%) quartile, and the median of each data set. Significant differences (*p* < 0.05) of each data set are labeled with lowercase letters.

The Richness and Shannon indices were evaluated to verify the accuracy of sequencing results. The Richness index of root tissues was higher in the NP group than in the SP and DP groups (Fig. 3A). Similarly, the Shannon index of root tissues was higher in the NP group than in the SP and DP groups (Fig. 3D). The Richness index of RS decreased from DP, NP, and SP in descending order (Fig. 3B), whereas the Shannon index of RS was prominently lower in the SP group than in the NP and DP groups (Fig. 3E). Interestingly, the Richness and Shannon indices of BS were remarkably higher in the SP group than in the DP and NP groups (Figs. 3C and 3F).

### Beta Diversity

**Fig. 4:**
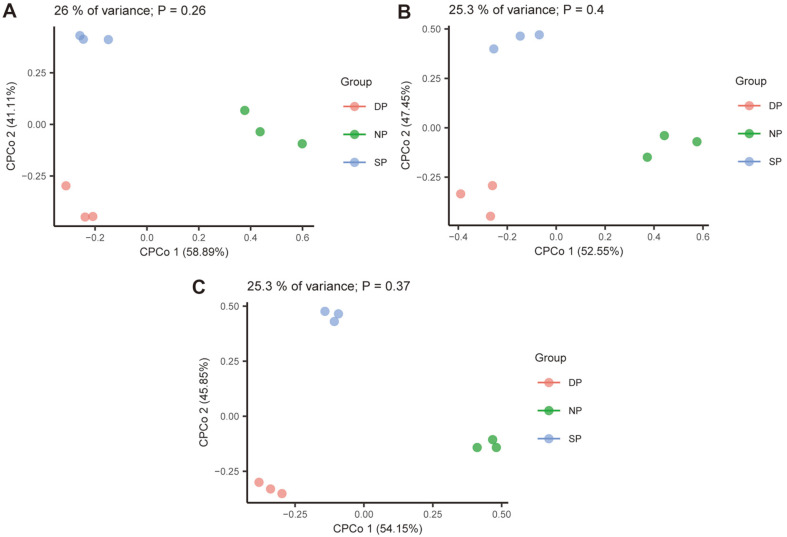
Constrained PCoA plot of Bray–Curtis distances of (**A**) RT, (**B**) RS, and (**C**) BS. Each point represents a different sample colored by the different groups of DP, NP, and SP. RT, root tissue; RS, rhizosphere soil; BS, bulk soil. NP, healthy plant; SP, diseased plant; DP, dead plant.

Constrained PCoA based on Bray–Curtis distance was used to compare β-diversity to assess the effect of the health status of *C. cathayensis* specimens on bacterial community composition. The results revealed differences in the bacterial community composition of RT samples among the DP, NP, and SP groups, which accounted for 26.0% of the variance (Fig. 4A, *p* = 0.26). Similarly, the RS and BS samples of the DP, NP, and SP groups showed the same differentiation, with both accounting for 25.3% of the variance (Fig. 4B and 4C).

### Bacterial Community Composition of Healthy, Diseased, and Dead *C. cathayensis* Specimens

**Fig. 5:**
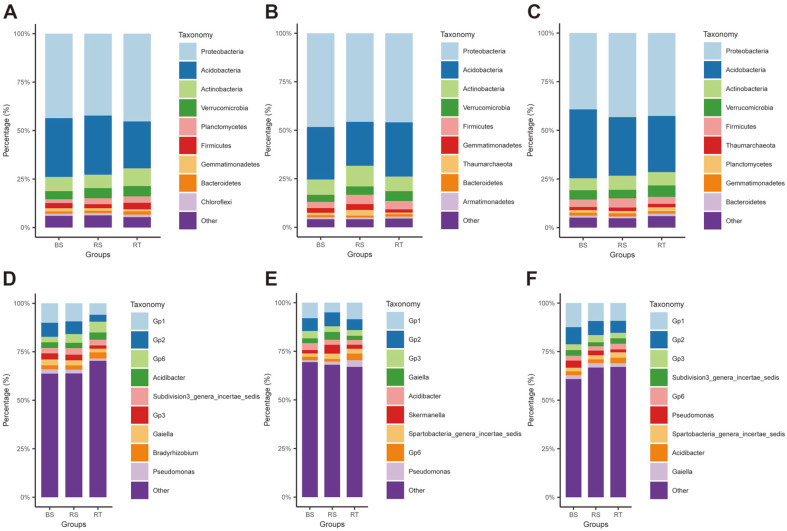
Relative abundances of the top 10 dominant bacteria at the phylum and genus levels in different groups of *C. cathayensis*. The top 10 abundant bacteria at the phylum in the (**A**) NP, (**B**) SP, and (**C**) DP *C. cathayensis*. The top 10 dominant bacteria at the genus in the (**D**) NP, (**E**) SP, and (**F**) DP *C. cathayensis*. NP, healthy plant; SP, diseased plant; DP, dead plant. BS, bulk soil; RS, rhizosphere soil; RT, root tissue.

To investigate the bacterial composition of healthy, diseased, and dead *C. cathayensis* specimens, we analyzed the top 10 bacteria at the phylum and genus levels. Samples in each of the NP, SP, and DP groups were divided into the RT, RS, and BS groups to analyze the bacterial community composition accurately. The results are shown in Fig. 5. In the NP group, the top 10 abundant bacterial phyla in RT, RS, and BS samples were Proteobacteria (42.26–45.30%), Acidobacteria (24.17–30.50%), Actinobacteria (6.92–9.13%), Verrucomicrobia (4.22–5.36%), Planctomycetes (1.92–3.28%), Firmicutes (2.08–3.37%), Gemmatimonadetes (1.06–1.64%), Bacteroidetes (1.09–1.66%), and Chloroflexi (1.23–1.33%). Although the top 10 bacteria were ranked consistently in RT, RS, and BS samples, the contents of some strains were different. For example, the abundance of Proteobacteria and Actinobacteria was higher in RT samples than in RS and BS samples, and that of Acidobacteria was higher in RS and BS samples than in RT samples (Fig. 5A). Similarly, the top genera found in RT, RS, and BS samples were *Gp1* (5.93–10.06%), *Gp2* (3.57–7.22%), *Gp6* (2.83–5.56%), *Acidibacter* (2.75–3.78%), *Subdivision3_genera_incertae_sedis* (2.76–3.39%), *Gp3* (1.65–3.17%), *Gaiella* (1.89–2.97%), *Bradyrhizobium* (2.09–3.16%), and *Pseudomonas* (1.24–2.22%) (Fig. 5D).

In the SP group, the top 10 phyla in RT, RS, and BS samples were Proteobacteria (45.67–48.33%), Acidobacteria (22.63–27.93%), Actinobacteria (7.55–10.64%), Verrucomicrobia (3.72–5.10%), Firmicutes (3.13–4.74%), Gemmatimonadetes (1.60–3.03%), Thaumarchaeota (0.85–2.90%), Bacteroidetes (0.95–1.23%), and Armatimonadetes (0.96–1.09%) (Fig. 5B). The top 10 bacterial genera in RT, RS, and BS samples were *Gp1* (4.99–8.52%), *Gp2* (5.58–7.16%), *Gp3* (2.94–3.80%), *Gaiella* (2.15–4.01%), *Acidibacter* (2.41–3.48%), *Skermanella* (1.75–4.55%), *Spartobacteria_genera_incertae_sedis* (1.68–2.73%), *Gp6* (1.33–3.41%), and *Pseudomonas* (0.95–3.42%) (Fig. 5E).

In the DP group, the top 10 dominant phyla in RT, RS, and BS samples were Proteobacteria (39.26–43.23%), Acidobacteria (28.87–35.40%), Actinobacteria (6.12–7.25%), Verrucomicrobia (4.44–5.88%), Firmicutes (3.28–4.65%), Thaumarchaeota (1.62–1.80%), Planctomycetes (1.23–2.01%), Gemmatimonadetes (1.16–1.56%), and Bacteroidetes (0.98–1.35%) (Fig. 5C). The top 10 genera in RT, RS, and BS samples were *Gp1* (9.16–12.42%), *Gp2* (6.18–8.83%), *Gp3* (2.80–3.72%), *Subdivision3_genera_incertae_sedis* (2.10–2.90%), *Gp6* (2.17–2.90%), *Pseudomonas* (1.48–3.63%), *Spartobacteria_genera_incertae_sedis* (1.85–2.77%), *Acidibacter* (2.41–3.48%), and *Gaiella* (1.94–2.35%) (Fig. 5F).

### Comparison of Bacterial Community Composition

**Fig. 6:**
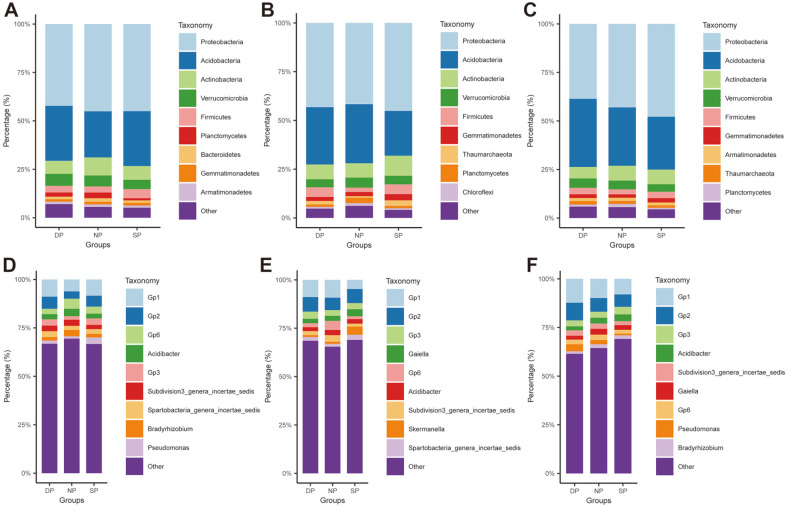
Relative abundances of the top 10 abundant bacteria at the phylum and genus levels in the samples of RT, RS, and BS. The top 10 dominant bacteria at the phylum in the samples of (**A**) RT, (**B**) RS, and (**C**) BS. The top 10 abundant bacteria at the genus in the samples of (**D**) RT, (**E**) RS, and (**F**) BS. RT, root tissue; RS, rhizosphere soil; BS, bulk soil. DP, dead plant; NP, healthy plant; SP, diseased plant.

The bacterial community composition was compared among healthy, diseased, and dead *C. cathayensis* specimens in the RT, RS, and BS groups (Fig. 6). In the RT group, the top 10 abundant phyla in healthy, diseased, and dead trees were Proteobacteria (42.27–45.10%), Acidobacteria (23.80–28.40%), Actinobacteria (6.75–9.33%), Verrucomicrobia (4.82–6.21%), Firmicutes (3.12–4.68%), Planctomycetes (1.11–2.93%), Bacteroidetes (1.27–1.81%), Gemmatimonadetes (1.23–1.36%), and Armatimonadetes (1.16–1.33%). The abundance of Acidobacteria was higher in dead and diseased trees (28.40% and 28.33%, respectively) than in healthy trees (23.80%), whereas that of Actinobacteria was higher in healthy trees (9.33%) than in dead and diseased trees (6.75% and 7.11%, respectively) (Fig. 6A). The dominant genera in healthy, diseased, and dead trees in the RT group were *Gp1*, *Gp2*, *Gp6*, *Acidibacter*, *Gp3*, *Subdivision3_genera_incertae_sedis*, *Spartobacteria_genera_incertae_sedis*, *Bradyrhizobium*, and *Pseudomonas* (Fig. 6D). The total relative abundance of the top nine bacterial genera in dead, healthy, and diseased trees in the RT group was 33.20, 30.55, and 33.34%, respectively. In particular, the abundance of *Gp1*, *Gp2*, and *Gp3* was higher in dead and diseased trees than in healthy trees, whereas that of *Gp6* and *Bradyrhizobium* was higher in healthy trees than in dead and diseased trees (Fig. 6D).

In the RS group, the top 10 dominant phyla in healthy, diseased, and dead trees were Proteobacteria, Acidobacteria, Actinobacteria, Verrucomicrobia, Firmicutes, Gemmatimonadetes, Thaumarchaeota, Planctomycetes, and Chloroflexi. The total relative abundance of the top nine bacterial phyla in dead, healthy, and diseased trees in the RS group was 95.29, 93.87, and 95.93%, respectively (Fig. 6B). At the genus level, *Gp1*, *Gp2*, *Gp3*, *Gaiella*, *Gp6*, *Acidibacter*, *Subdivision3_genera_incertae_sedis*, *Skermanella*, and *Spartobacteria_genera_incertae_sedis* were the most abundant bacteria in dead, healthy, and diseased trees in the RS group. In particular, the abundance of *Skermanella* was higher in diseased trees (4.19%), whereas that of *Gp6* was higher in healthy trees (4.65%) (Fig. 6E).

In the BS group, Proteobacteria, Acidobacteria, Actinobacteria, Verrucomicrobia, Firmicutes, Gemmatimonadetes, Armatimonadetes, Thaumarchaeota, and Planctomycetes were the top 10 dominant phyla in dead, healthy, and diseased trees (Fig. 6C). At the genus level, the most abundant bacteria in dead, healthy, and diseased trees in the BS group were *Gp1*, *Gp2*, *Gp3*, *Acidibacter*, *Subdivision3_genera_incertae_sedis*, *Gaiella*, *Gp6*, *Pseudomonas*, and *Bradyrhizobium*. The total relative abundance of the top nine bacterial genera in dead, healthy, and diseased trees in the RS group was 38.55, 35.59, and 30.90%, respectively (Fig. 6F).

### Variance of Bacterial Communities in Diseased and Dead *C. cathayensis* Specimens

**Fig. 7:**
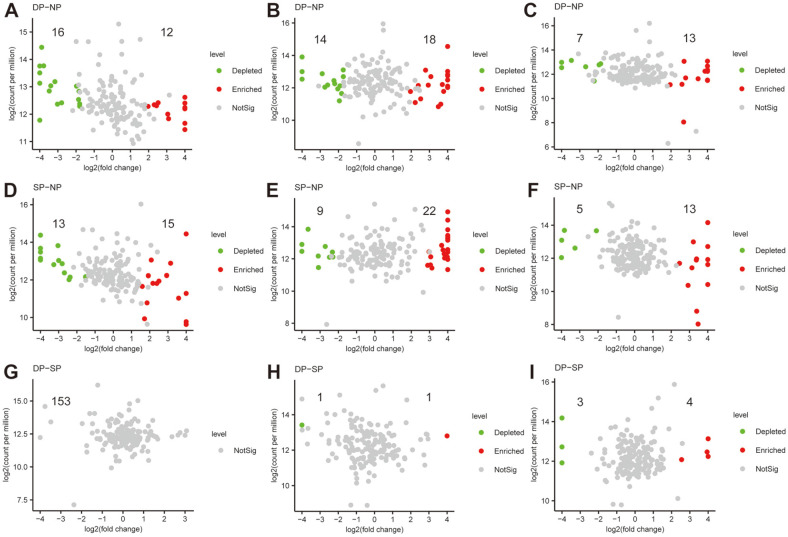
Volcano plot of differential bacterial abundance in the samples of RT, RS, and BS. The bacterial community changes in the (**A**) RT, (**B**) RS, and (**C**) BS samples of DP *C. cathayensis* compared to the NP *C. cathayensis*. The bacterial community changes in the (**D**) RT, (**E**) RS, and (**F**) BS samples of SP *C. cathayensis* compared to the NP *C. cathayensis*. The bacterial community changes in the (**G**) RT, (**H**) RS, and (**I**) BS samples of DP *C. cathayensis* compared to SP *C. cathayensis*. RT, root tissue; RS, rhizosphere soil; BS, bulk soil. DP, dead plant; NP, healthy plant; SP, diseased plant. fold-change > 2, *p* < 0.01. Green points are significantly depleted bacteria, while red points are significantly enriched bacteria.

To verify the variance of bacterial communities in diseased and dead *C. cathayensis* specimens, we compared the bacterial community composition in these trees at RT, RS, and BS levels (Fig. 7 and Tables S1–S3). In the RT group, the abundance of 16 bacterial genera was significantly lower and that of 12 bacterial genera was higher in dead trees than in healthy trees (Fig. 7A and Table S1) (*p* < 0.01). Similarly, the abundance of 13 bacterial genera was lower and that of 15 bacterial genera was higher in diseased trees than in healthy trees (Fig. 7D and Table S1). Overall, no significant differences were observed in bacterial community composition between dead and diseased *C. cathayensis* specimens (Fig. 7G and Table S1).

In the RS group, the abundance of 14 bacterial genera was lower and that of 18 bacterial genera was higher in dead *C. cathayensis* than in healthy trees (Fig. 7B and Table S2). The abundance of 9 bacterial genera was significantly lower and that of 22 bacterial genera was higher in diseased trees than in healthy trees (Fig. 7E and Table S2). In addition, the abundance of one genus was lower and that of another genus was higher in dead trees than in diseased trees (Fig. 7H and Table S2).

In the BS group, the abundance of 7 bacterial genera was lower and that of 13 bacterial genera was significantly higher in dead *C. cathayensis* than in healthy trees (Fig. 7C and Table S3). The abundance of 5 bacterial genera was lower and that of 13 bacterial genera was higher in diseased trees than in healthy trees (Fig. 7F and Table S3). Similarly, the abundance of 3 bacterial genera was lower and that of 4 bacterial genera was higher in dead trees than in diseased trees (Fig. 7I and Table S3).

**Fig. 8:**
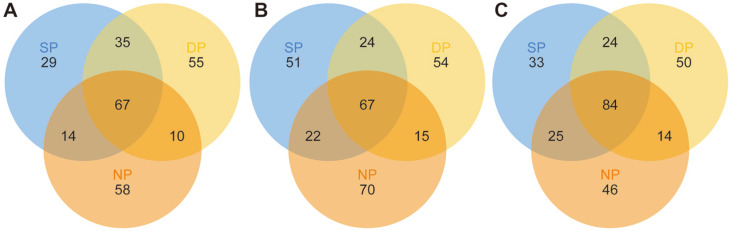
Venn diagrams of bacteria in the samples of (**A**) RT, (**B**) RS, and (**C**) BS from different healthy *C. cathayensis* specimens. RT, root tissue; RS, rhizosphere soil; BS, bulk soil. DP, dead plant; NP, healthy plant; SP, diseased plant.

Finally, we analyzed the common bacterial communities among healthy, diseased, and dead *C. cathayensis* specimens. The results are shown in Fig. 8 and Tables S4–S6. In the RT group, a total of 67 bacterial genera were common among the dead, healthy, and diseased trees. In particular, 35 bacterial genera were common between the dead and diseased trees (Fig. 8A and Table S4). Similarly, a total of 67 bacterial genera were common among the dead, healthy, and diseased *C. cathayensis* specimens in the RS group. A total of 24 bacterial genera were common between the dead and diseased trees in the RS group (Fig. 8B and Table S5). In the BS group, 24 bacterial genera were common between the diseased and dead trees. In addition, 84 bacterial genera were common among the dead, healthy, and diseased *C. cathayensis* in the BS group (Fig. 8C and Table S6).

### Distinct Core Genera in Healthy, Diseased, and Dead *C. cathayensis* Specimens

**Fig. 9:**
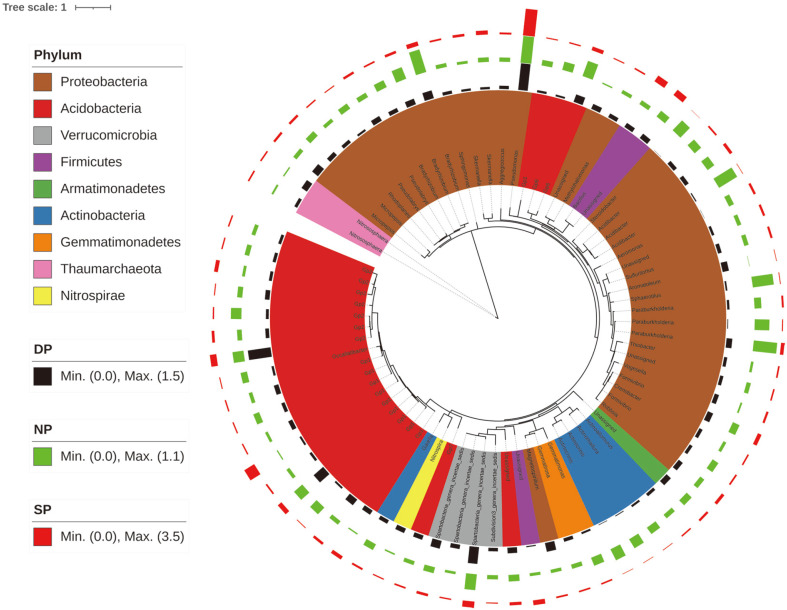
Relative abundances of the top abundant bacteria in the root tissue of different healthy *C. cathayensis* specimens. A taxonomic tree showing the core bacterial communities of different healthy *C. cathayensis* specimens. Color ranges show phyla within the tree. Red, green, and black of colored bars display the relative abundance of each ASV from SP, NP, and DP of *C. cathayensis*, respectively. The taxonomic dendrogram was drawn by iTOL. DP, dead plant; NP, healthy plant; SP, diseased plant.

To provide a complete overview of distinct core genera in healthy, diseased, and dead *C. cathayensis* specimens in the RT group, the top 75 bacterial genera were visualized on a phylogenetic tree (Fig. 9). The abundance of *Acidibacter*, *Actinocrinis*, *Actinomadura*, *Bradyrhizobium*, *Paraburkholderia*, *Gp6*, *Sphaerotilus*, and *Steroidobacter* was higher in healthy *C. cathayensis* than in diseased and dead trees. In addition, the abundance of *Aromatoleum*, *Gp1*, *Gp2*, *Magnetospirillum*, and *Nitrososphaera* was higher in diseased and dead trees than in healthy ones.

## Discussion

In this study, *16S rRNA* amplicon sequencing revealed differences in bacterial community composition among healthy, diseased, and dead specimens of *C. cathayensis*. Proteobacteria, Acidobacteria, and Actinobacteria were found to be the most dominant bacterial phyla in the root tissue, rhizosphere soil, and bulk soil, accounting for approximately 80.32% of the total bacterial communities (Fig. 5A–5C). The abundance of Acidobacteria and Actinobacteria has been reported to be high in soils worldwide [28], and these bacteria are adequately adapted to environmental stresses and poor soils [29]. Therefore, the results of this study are consistent with those of previous studies. Furthermore, we found differences in the abundance of Acidobacteria and Actinobacteria among healthy, diseased, and dead *C. cathayensis* specimens. The root tissues of healthy trees contained more Actinobacteria than those of diseased and dead trees (Fig. 6A). Lee *et al*. reported that the abundance of Actinobacteria and Firmicutes was significantly lower in diseased RS than in healthy RS. In addition, they verified that artificial disruption in the balance of bacterial species (dysbiosis) increased the incidence of wilt in tomato plants [30], indicating that dysbiosis can cause plant diseases [30-34]. Therefore, dysbiosis may be associated with the development of root rot in *C. cathayensis*.

Furthermore, we analyzed the dominant bacterial species in the root tissues of healthy, diseased, and dead *C. cathayensis* specimens. As shown in Fig. 9, the abundance of four genera belonging to the Actinobacteria phylum, namely, *Actinoallomurus*, *Actinomadura*, *Actinocrinis*, and *Gaiella*, was significantly higher in the root tissues of healthy *C. cathayensis* than in those of diseased and dead trees. Yadav *et al*. reported that Actinobacteria can colonize plant roots and produce antibiotics to inhibit the growth of plant pathogens [29]. Therefore, Actinobacteria may help *C. cathayensis* resist the growth of pathogenic bacteria.

Another important finding of this study was that the abundance of *Acidibacter*, *Bradyrhizobium*, *Paraburkholderia*, *Sphaerotilus*, and *Steroidobacter* belonging to the Proteobacteria phylum was higher in the root tissues of healthy *C. cathayensis* specimens than in those of diseased and dead trees (Fig. 9). We have previously demonstrated that the available phosphorus content is lower in the soil of diseased *C. cathayensis* specimens than in those of healthy trees [35]. Similarly, Fu *et al*. reported that the abundance of *Acidibacter* was significantly associated with the content of total and available phosphorus in tea garden soil [36]. Therefore, *Acidibacter* can be used as an indicator of soil nutrients. *Bradyrhizobium* [37] and *Paraburkholderia* [38] exert beneficial effects on host plants. For instance, *Bradyrhizobium*, a nitrogen-fixing symbiont of soybean, is very important for the healthy growth of soybeans [37]. Therefore, *Acidibacter*, *Bradyrhizobium*, *Paraburkholderia*, *Sphaerotilus*, and *Steroidobacter* belonging to the Proteobacteria phylum are beneficial microbes found in the rhizosphere of *C. cathayensis*. Overall, the occurrence of root rot in *C. cathayensis* is closely related to dysbiosis of the root microbiota, especially a reduction in the abundance of some beneficial microbes.

## Supplemental Materials

Supplementary data for this paper are available on-line only at http://jmb.or.kr.
