# Supplementary material for: Bacterial Microbiome Differences between the Roots of Diseased and Healthy Chinese Hickory (Carya cathayensis) Trees
Source: J Microbiol Biotechnol. 2023 Jul 21;33(10):1299–308. doi: 10.4014/jmb.2304.04054 (PMC10619558; doi:10.4014/jmb.2304.04054)
Supplement: Supplementary file 1 [file jmb-33-10-1299-supple.pdf]

J. Microbiol. Biotechnol.
https://doi.org/10.4014/jmb.2304.04054

Differentially changed bacteria in the root tissue of DP *C. cathayensis* trees aganist NP *C. cathayensis* trees

ASV_ID	log2FC	log2CPM	PValue	level
ASV_72	-9.814	13.131	3.87E-07	Depleted
ASV_82	-6.503	11.779	0.006980676	Depleted
ASV_40	-5.002	13.508	0.00021532	Depleted
ASV_26	-4.783	13.759	0.000193887	Depleted
ASV_12	-3.896	14.441	0.006200998	Depleted
ASV_19	-3.785	13.765	0.008631981	Depleted
ASV_52	-3.477	12.846	0.001478007	Depleted
ASV_63	-3.393	13.035	0.004136643	Depleted
ASV_69	-3.164	13.187	0.002738214	Depleted
ASV_140	-3.024	12.367	0.000919078	Depleted
ASV_135	-2.796	12.415	0.004605559	Depleted
ASV_35	-1.975	13.024	0.007728809	Depleted
ASV_88	-1.86	12.534	0.032738256	Depleted
ASV_59	-1.837	12.835	0.018709021	Depleted
ASV_143	-1.8	12.291	0.034649074	Depleted
ASV_118	-1.799	12.373	0.028453906	Depleted
ASV_39	1.971	12.284	0.026261836	Enriched
ASV_70	2.31	12.343	0.012809386	Enriched
ASV_24	2.437	12.308	0.004089069	Enriched
ASV_57	2.526	12.418	0.002285315	Enriched
ASV_132	3.056	12.002	0.00582956	Enriched
ASV_123	3.102	11.833	0.00324849	Enriched
ASV_127	4.265	11.667	7.85E-05	Enriched
ASV_84	4.812	12.616	0.001834466	Enriched
ASV_100	4.882	12.24	0.005006498	Enriched
ASV_83	6.912	12.403	0.000178292	Enriched
ASV_110	7.789	11.438	4.91E-05	Enriched
ASV_113	10.627	12.196	7.65E-06	Enriched

Differentially changed bacteria in the root tissue of SP *C. cathayensis* trees aganist NP *C. cathayensis* trees

ASV_ID	log2FC	log2CPM	PValue	level
ASV_72	-7.538	13.144	4.37E-05	Depleted
ASV_40	-4.416	13.475	0.000994469	Depleted
ASV_69	-4.389	13.05	1.13E-05	Depleted
ASV_19	-4.228	13.677	0.001275473	Depleted
ASV_12	-4.057	14.377	0.008017962	Depleted
ASV_52	-3.268	12.805	0.003467475	Depleted
ASV_26	-3.039	13.818	0.013391301	Depleted
ASV_63	-3.001	13.024	0.0214251	Depleted
ASV_49	-2.808	12.854	0.017018295	Depleted
ASV_140	-2.686	12.366	0.001137287	Depleted
ASV_102	-2.431	12.016	0.002134657	Depleted
ASV_143	-2.374	12.139	0.001343508	Depleted
ASV_119	-1.523	12.161	0.012449073	Depleted
ASV_206	1.591	11.655	0.028927609	Enriched
ASV_84	1.713	9.929	0.014092129	Enriched
ASV_123	1.853	10.777	0.013564141	Enriched
ASV_39	1.922	12.22	0.01471106	Enriched
ASV_33	2.084	13.05	0.029176115	Enriched
ASV_129	2.172	11.82	0.019323089	Enriched
ASV_126	2.387	11.814	0.000632813	Enriched
ASV_86	2.505	11.938	0.024832827	Enriched
ASV_93	2.93	12.232	0.01059008	Enriched
ASV_24	3.145	12.88	0.000612086	Enriched
ASV_127	3.587	11.028	2.46E-05	Enriched
ASV_83	4.077	9.772	3.33E-06	Enriched
ASV_7	5.138	14.444	0.000867264	Enriched
ASV_110	7.664	11.281	1.08E-05	Enriched
ASV_113	7.977	9.627	6.89E-10	Enriched

Differentially changed bacteria in the root tissue of DP *C. cathayensis* trees aganist SP *C. cathayensis* trees

ASV_ID	log2FC	log2CPM	PValue	level
		none		

Differentially changed bacteria in the rhizosphere soil of DP *C. cathayensis* trees aganist NP *C. cathayensis* trees

ASV_ID	log2FC	log2CPM	PValue	level
ASV_146	-5.294	12.536	0.001694469	Depleted
ASV_99	-5.227	13.001	1.23E-03	Depleted
ASV_22	-4.616	13.905	1.09E-03	Depleted
ASV_80	-2.893	12.872	0.015348008	Depleted
ASV_129	-2.747	12.044	0.016728093	Depleted
ASV_108	-2.594	12.173	0.022365248	Depleted
ASV_70	-2.248	12.445	0.013774876	Depleted
ASV_117	-2.206	12.255	0.012929993	Depleted
ASV_141	-2.038	11.938	0.040966457	Depleted
ASV_126	-1.95	11.199	0.037424793	Depleted
ASV_109	-1.917	12.123	0.025349602	Depleted
ASV_101	-1.829	11.65	0.036297634	Depleted
ASV_68	-1.738	13.104	0.027684924	Depleted
ASV_55	-1.734	12.692	0.037858338	Depleted
ASV_156	1.957	11.774	0.033194832	Enriched
ASV_123	2.219	11.093	0.007295585	Enriched
ASV_132	2.388	12.144	0.034675607	Enriched
ASV_98	2.518	11.32	0.040097886	Enriched
ASV_46	2.788	13.091	0.006258399	Enriched
ASV_53	2.936	12.167	0.029488351	Enriched
ASV_84	3.079	12.693	0.003855926	Enriched
ASV_163	3.49	10.84	0.009136144	Enriched
ASV_41	3.624	10.993	0.006321061	Enriched
ASV_67	3.709	12.205	0.00131571	Enriched
ASV_72	3.781	11.78	0.001257596	Enriched
ASV_54	4.394	12.748	0.002831306	Enriched
ASV_36	4.493	12.814	0.004419184	Enriched
ASV_157	4.869	12.105	0.000166675	Enriched
ASV_3	4.922	14.552	0.001802651	Enriched
ASV_113	5.423	13.016	0.000404768	Enriched
ASV_28	5.522	12.023	0.002488078	Enriched
ASV_74	6.623	12.485	2.03E-09	Enriched

Differentially changed bacteria in the rhizosphere soil of SP *C. cathayensis* trees aganist NP *C. cathayensis* trees

ASV_ID	log2FC	log2CPM	PValue	level
ASV_99	-4.942	12.896	0.00352207	Depleted
ASV_146	-4.133	12.465	0.011458919	Depleted
ASV_22	-3.661	13.844	0.019213885	Depleted
ASV_75	-3.103	12.173	0.025083289	Depleted
ASV_120	-3.091	11.457	0.016270915	Depleted
ASV_80	-2.718	12.77	0.031866718	Depleted
ASV_108	-2.489	12.099	0.033023439	Depleted
ASV_21	-2.325	12.42	0.036237152	Depleted
ASV_117	-2.299	12.115	0.035458673	Depleted
ASV_538	2.898	11.594	0.012265627	Enriched
ASV_97	2.974	12.442	0.031557261	Enriched
ASV_123	3.054	11.62	0.018925369	Enriched
ASV_53	3.067	12.124	0.028758124	Enriched
ASV_54	3.133	11.426	0.024664362	Enriched
ASV_43	3.665	12.839	0.012921139	Enriched
ASV_64	3.742	12.551	0.006076435	Enriched
ASV_98	3.805	12.308	0.019982457	Enriched
ASV_67	3.828	12.049	0.004114276	Enriched
ASV_36	3.976	12.113	0.024394451	Enriched
ASV_77	4.031	13.159	0.015562251	Enriched
ASV_69	4.122	13.228	0.007779468	Enriched
ASV_112	4.3	12.414	0.00482735	Enriched
ASV_5	4.3	14.927	0.010276022	Enriched
ASV_20	4.479	13.813	0.020682521	Enriched
ASV_72	4.813	12.574	0.00844161	Enriched
ASV_163	4.86	11.963	0.003656261	Enriched
ASV_3	4.981	14.412	0.007415283	Enriched
ASV_74	5.567	11.329	0.002946529	Enriched
ASV_47	5.97	13.456	0.002700677	Enriched
ASV_41	6.273	13.365	0.008418479	Enriched
ASV_28	6.934	13.259	0.006077521	Enriched

Differentially changed bacteria in the rhizosphere soil of DP *C. cathayensis* trees aganist SP *C. cathayensis* trees

ASV_ID	log2FC	log2CPM	PValue	level
ASV_47	-6.901	13.417	0.002118158	Depleted
ASV_113	8.189	12.804	7.70E-06	Enriched

Differentially changed bacteria in the bulk soil of DP *C. cathayensis* trees aganist NP *C. cathayensis* trees

ASV_ID	log2FC	log2CPM	PValue	level
ASV_97	-4.689	12.981	5.51E-04	Depleted
ASV_132	-4.51	12.545	2.13E-04	Depleted
ASV_82	-3.465	13.139	0.018547366	Depleted
ASV_44	-2.681	12.617	0.00216716	Depleted
ASV_181	-2.212	11.444	0.01064385	Depleted
ASV_28	-1.946	12.776	0.015666764	Depleted
ASV_41	-1.858	12.862	0.016553677	Depleted
ASV_113	1.953	11.138	0.015704848	Enriched
ASV_86	2.601	11.17	0.000531948	Enriched
ASV_12	2.693	8.057	0.020388665	Enriched
ASV_18	2.715	13.066	0.006280586	Enriched
ASV_116	2.802	11.696	0.002192891	Enriched
ASV_89	3.485	11.626	0.005346858	Enriched
ASV_49	3.862	12.25	0.003301063	Enriched
ASV_146	4.585	12.239	0.006276939	Enriched
ASV_57	5.619	12.674	0.0003967	Enriched
ASV_102	5.952	11.516	0.00030773	Enriched
ASV_144	5.953	11.486	1.07E-05	Enriched
ASV_73	7.637	12.355	7.49E-05	Enriched
ASV_38	9.237	13.079	1.93E-05	Enriched

Differentially changed bacteria in the bulk soil of SP *C. cathayensis* trees aganist NP *C. cathayensis* trees

ASV_ID	log2FC	log2CPM	PValue	level
ASV_215	-4.548	12.038	0.009836034	Depleted
ASV_97	-3.989	13.091	0.003952087	Depleted
ASV_45	-3.84	13.688	0.003851047	Depleted
ASV_132	-3.261	12.613	0.004953942	Depleted
ASV_14	-2.091	13.664	0.017957256	Depleted
ASV_113	2.454	11.69	0.006884705	Enriched
ASV_57	2.922	10.369	0.005634534	Enriched
ASV_89	3.134	11.417	0.011086334	Enriched
ASV_35	3.211	12.99	0.015429988	Enriched
ASV_49	3.375	11.889	0.001672106	Enriched
ASV_86	3.388	11.958	0.000606778	Enriched
ASV_73	3.395	8.804	0.001309041	Enriched
ASV_38	3.469	8.025	0.005528056	Enriched
ASV_144	4.687	10.421	7.74E-05	Enriched
ASV_102	6.083	11.616	8.80E-06	Enriched
ASV_12	9.362	14.159	1.44E-08	Enriched
ASV_71	9.57	12.709	4.56E-08	Enriched
ASV_173	10.65	11.919	6.95E-07	Enriched

Differentially changed bacteria in the bulk soil of DP *C. cathayensis* trees aganist SP *C. cathayensis* trees

ASV_ID	log2FC	log2CPM	PValue	level
ASV_173	-8.866	11.917	5.09E-06	Depleted
ASV_12	-6.727	14.181	1.05E-05	Depleted
ASV_71	-6.355	12.722	0.000494878	Depleted
ASV_90	2.548	12.079	0.006040009	Enriched
ASV_73	3.943	12.467	0.00419804	Enriched
ASV_146	5.584	12.239	0.000425854	Enriched
ASV_38	5.679	13.133	0.001564541	Enriched

The shared strains from the root tissue of NP, SP, and DP *C. cathayensis* trees

OTU	Kingdom	Phylum	Class	Order	Family	Genus	Species
ASV_1	Bacteria	Acidobacteria	Acidobacteria_Gp2	Unassigned	Unassigned	Gp2	Unassigned
ASV_2	Bacteria	Proteobacteria	Gammaproteobacteria	Pseudomonadales	Pseudomonadaceae	Pseudomonas	Unassigned
ASV_3	Bacteria	Proteobacteria	Alphaproteobacteria	Rhizobiales	Bradyrhizobiaceae	Bradyrhizobium	Unassigned
ASV_4	Bacteria	Proteobacteria	Alphaproteobacteria	Rhizobiales	Bradyrhizobiaceae	Bradyrhizobium	Unassigned
ASV_5	Bacteria	Acidobacteria	Acidobacteria_Gp1	Unassigned	Unassigned	Gp1	Unassigned
ASV_6	Bacteria	Proteobacteria	Betaproteobacteria	Burkholderiales	Burkholderiaceae	Paraburkholderia	Unassigned
ASV_8	Bacteria	Acidobacteria	Acidobacteria_Gp6	Unassigned	Unassigned	Gp6	Unassigned
ASV_9	Bacteria	Firmicutes	Bacilli	Bacillales	Unassigned	Unassigned	Unassigned
ASV_11	Bacteria	Firmicutes	Bacilli	Bacillales	Bacillaceae_1	Bacillus	Unassigned
ASV_13	Bacteria	Proteobacteria	Alphaproteobacteria	Rhizobiales	Bradyrhizobiaceae	Bradyrhizobium	Unassigned
ASV_14	Bacteria	Proteobacteria	Betaproteobacteria	Neisseriales	Chromobacteriaceae	Formivibrio	Unassigned
ASV_15	Bacteria	Proteobacteria	Gammaproteobacteria	Gammaproteobacteria_incertae_sedis	Unassigned	Acidibacter	Unassigned
ASV_16	Bacteria	Acidobacteria	Unassigned	Unassigned	Unassigned	Unassigned	Unassigned
ASV_17	Bacteria	Armatimonadetes	Unassigned	Unassigned	Unassigned	Unassigned	Unassigned
ASV_18	Bacteria	Acidobacteria	Acidobacteria_Gp1	Unassigned	Unassigned	Gp1	Unassigned
ASV_20	Bacteria	Proteobacteria	Betaproteobacteria	Burkholderiales	Burkholderiaceae	Robbsia	Unassigned
ASV_21	Bacteria	Verrucomicrobia	Spartobacteria	Unassigned	Unassigned	Spartobacteria_genera_incertae_sedis	Unassigned
ASV_22	Bacteria	Proteobacteria	Alphaproteobacteria	Rhodospirillales	Rhodospirillaceae	Magnetospirillum	Unassigned
ASV_25	Bacteria	Proteobacteria	Alphaproteobacteria	Rhodospirillales	Azospirillaceae	Skermanella	Unassigned
ASV_27	Bacteria	Proteobacteria	Gammaproteobacteria	Gammaproteobacteria_incertae_sedis	Unassigned	Acidibacter	Unassigned
ASV_28	Bacteria	Proteobacteria	Alphaproteobacteria	Micropepsales	Micropepsaceae	Micropepsis	Unassigned
ASV_29	Bacteria	Proteobacteria	Alphaproteobacteria	Micropepsales	Micropepsaceae	Micropepsis	Unassigned
ASV_30	Bacteria	Acidobacteria	Acidobacteria_Gp2	Unassigned	Unassigned	Gp2	Unassigned
ASV_31	Bacteria	Acidobacteria	Acidobacteria_Gp2	Unassigned	Unassigned	Gp2	Unassigned
ASV_32	Bacteria	Actinobacteria	Thermoleophilia	Gaiellales	Gaiellaceae	Gaiella	Unassigned
ASV_33	Bacteria	Acidobacteria	Acidobacteria_Gp2	Unassigned	Unassigned	Gp2	Unassigned
ASV_34	Bacteria	Proteobacteria	Alphaproteobacteria	Rhizobiales	Bradyrhizobiaceae	Bradyrhizobium	Unassigned
ASV_35	Bacteria	Gemmatimonadetes	Gemmatimonadetes	Gemmatimonadales	Gemmatimonadaceae	Gemmatimonas	Unassigned
ASV_36	Bacteria	Proteobacteria	Betaproteobacteria	Burkholderiales	Burkholderiaceae	Paraburkholderia	Unassigned
ASV_37	Bacteria	Proteobacteria	Betaproteobacteria	Nitrosomonadales	Thiobacillaceae	Sulfuritortus	Unassigned
ASV_41	Bacteria	Acidobacteria	Acidobacteria_Gp7	Unassigned	Unassigned	Gp7	Unassigned
ASV_42	Bacteria	Proteobacteria	Betaproteobacteria	Rhodocyclales	Zoogloeaceae	Unassigned	Unassigned
ASV_43	Bacteria	Proteobacteria	Gammaproteobacteria	Chromatiales	Ectothiorhodospiraceae	Methylohalomonas	Unassigned
ASV_44	Bacteria	Acidobacteria	Acidobacteria_Gp1	Unassigned	Unassigned	Gp1	Unassigned
ASV_45	Bacteria	Gemmatimonadetes	Gemmatimonadetes	Gemmatimonadales	Gemmatimonadaceae	Gemmatirosa	Unassigned
ASV_46	Bacteria	Acidobacteria	Acidobacteria_Gp2	Unassigned	Unassigned	Gp2	Unassigned
ASV_48	Bacteria	Acidobacteria	Acidobacteria_Gp1	Unassigned	Unassigned	Occallatibacter	Unassigned
ASV_50	Bacteria	Acidobacteria	Acidobacteria_Gp1	Unassigned	Unassigned	Gp1	Unassigned
ASV_51	Bacteria	Actinobacteria	Actinobacteria	Streptosporangiales	Thermomonosporaceae	Actinoallomurus	Unassigned
ASV_53	Bacteria	Acidobacteria	Acidobacteria_Gp2	Unassigned	Unassigned	Gp2	Unassigned
ASV_54	Bacteria	Proteobacteria	Betaproteobacteria	Neisseriales	Neisseriaceae	Crenobacter	Unassigned
ASV_55	Bacteria	Acidobacteria	Acidobacteria_Gp1	Unassigned	Unassigned	Gp1	Unassigned
ASV_58	Bacteria	Proteobacteria	Alphaproteobacteria	Sphingomonadales	Sphingomonadaceae	Sphingomonas	Unassigned
ASV_60	Bacteria	Acidobacteria	Acidobacteria_Gp2	Unassigned	Unassigned	Gp2	Unassigned
ASV_61	Bacteria	Proteobacteria	Alphaproteobacteria	Rhizobiales	Bradyrhizobiaceae	Rhodoplanes	Unassigned
ASV_62	Bacteria	Acidobacteria	Acidobacteria_Gp1	Unassigned	Unassigned	Gp1	Unassigned
ASV_65	Bacteria	Acidobacteria	Acidobacteria_Gp1	Unassigned	Unassigned	Gp1	Unassigned
ASV_66	Bacteria	Proteobacteria	Alphaproteobacteria	Rhizobiales	Bradyrhizobiaceae	Pseudolabrys	Unassigned
ASV_71	Bacteria	Acidobacteria	Acidobacteria_Gp1	Unassigned	Unassigned	Gp1	Unassigned
ASV_78	Bacteria	Proteobacteria	Gammaproteobacteria	Gammaproteobacteria_incertae_sedis	Unassigned	Acidibacter	Unassigned
ASV_73	Bacteria	Proteobacteria	Betaproteobacteria	Burkholderiales	Burkholderiaceae	Unassigned	Unassigned
ASV_74	Bacteria	Proteobacteria	Betaproteobacteria	Neisseriales	Chromobacteriaceae	Vogesella	Unassigned
ASV_75	Bacteria	Proteobacteria	Betaproteobacteria	Neisseriales	Chromobacteriaceae	Formivibrio	Unassigned
ASV_79	Bacteria	Proteobacteria	Alphaproteobacteria	Rhizobiales	Bradyrhizobiaceae	Pseudolabrys	Unassigned
ASV_89	Bacteria	Proteobacteria	Alphaproteobacteria	Rhizobiales	Roseiarcaceae	Roseiarcus	Unassigned
ASV_91	Bacteria	Verrucomicrobia	Subdivision3	Unassigned	Unassigned	Subdivision3_genera_incertae_sedis	Unassigned
ASV_96	Bacteria	Proteobacteria	Alphaproteobacteria	Rhodospirillales	Unassigned	Unassigned	Unassigned
ASV_99	Bacteria	Acidobacteria	Acidobacteria_Gp1	Unassigned	Unassigned	Gp1	Unassigned
ASV_105	Bacteria	Unassigned	Unassigned	Unassigned	Unassigned	Unassigned	Unassigned
ASV_108	Bacteria	Acidobacteria	Acidobacteria_Gp1	Unassigned	Unassigned	Gp1	Unassigned
ASV_115	Bacteria	Acidobacteria	Acidobacteria_Gp6	Unassigned	Unassigned	Gp6	Unassigned
ASV_117	Bacteria	Actinobacteria	Thermoleophilia	Gaiellales	Gaiellaceae	Gaiella	Unassigned
ASV_125	Bacteria	Proteobacteria	Alphaproteobacteria	Rhodospirillales	Reyranellaceae	Reyranella	Unassigned
ASV_136	Bacteria	Proteobacteria	Alphaproteobacteria	Rhizobiales	Bradyrhizobiaceae	Variibacter	Unassigned
ASV_158	Bacteria	Proteobacteria	Alphaproteobacteria	Rhizobiales	Bradyrhizobiaceae	Bradyrhizobium	Unassigned
ASV_450	Bacteria	Proteobacteria	Alphaproteobacteria	Rhodospirillales	Azospirillaceae	Skermanella	Unassigned
ASV_2451	Bacteria	Proteobacteria	Deltaproteobacteria	Myxococcales	Myxococcaceae	Aggregicoccus	Unassigned

The shared strains from the root tissue of SP and DP *C. cathayensis* trees

OTU	Kingdom	Phylum	Class	Order	Family	Genus	Species
ASV_7	Bacteria	Acidobacteria	Acidobacteria_Gp1	Unassigned	Unassigned	Gp1	Unassigned
ASV_24	Bacteria	Acidobacteria	Acidobacteria_Gp1	Unassigned	Unassigned	Gp1	Unassigned
ASV_38	Archaea	Thaumarchaeota	Unassigned	Nitrososphaerales	Nitrososphaeraceae	Nitrososphaera	Unassigned
ASV_39	Bacteria	Proteobacteria	Betaproteobacteria	Neisseriales	Chromobacteriaceae	Vogesella	Unassigned
ASV_56	Bacteria	Verrucomicrobia	Spartobacteria	Unassigned	Unassigned	Spartobacteria_genera_incertae_sedis	Unassigned
ASV_57	Bacteria	Proteobacteria	Gammaproteobacteria	Unassigned	Unassigned	Unassigned	Unassigned
ASV_64	Bacteria	Proteobacteria	Betaproteobacteria	Burkholderiales	Burkholderiales_incertae_sedis	Thiobacter	Unassigned
ASV_68	Bacteria	Proteobacteria	Betaproteobacteria	Nitrosomonadales	Sterolibacteriaceae	Sterolibacterium	Unassigned
ASV_70	Bacteria	Nitrospirae	Nitrospira	Nitrospirales	Nitrospiraceae	Nitrospira	Unassigned
ASV_76	Bacteria	Proteobacteria	Betaproteobacteria	Rhodocyclales	Zoogloeaceae	Thauera	Unassigned
ASV_77	Archaea	Thaumarchaeota	Unassigned	Nitrososphaerales	Nitrososphaeraceae	Nitrososphaera	Unassigned
ASV_80	Bacteria	Acidobacteria	Acidobacteria_Gp2	Unassigned	Unassigned	Gp2	Unassigned
ASV_86	Bacteria	Proteobacteria	Unassigned	Unassigned	Unassigned	Unassigned	Unassigned
ASV_90	Bacteria	Acidobacteria	Acidobacteria_Gp2	Unassigned	Unassigned	Gp2	Unassigned
ASV_92	Bacteria	Gemmatimonadetes	Gemmatimonadetes	Gemmatimonadales	Gemmatimonadaceae	Gemmatimonas	Unassigned
ASV_94	Bacteria	Proteobacteria	Alphaproteobacteria	Rhodospirillales	Rhodospirillaceae	Ferruginivarius	Unassigned
ASV_98	Bacteria	Proteobacteria	Alphaproteobacteria	Rhodospirillales	Azospirillaceae	Unassigned	Unassigned
ASV_101	Bacteria	Proteobacteria	Gammaproteobacteria	Pseudomonadales	Moraxellaceae	Perlucidibaca	Unassigned
ASV_107	Bacteria	Proteobacteria	Alphaproteobacteria	Caulobacterales	Caulobacteraceae	Phenylobacterium	Unassigned
ASV_110	Bacteria	Proteobacteria	Betaproteobacteria	Neisseriales	Neisseriaceae	Aquaphilus	Unassigned
ASV_112	Bacteria	Acidobacteria	Acidobacteria_Gp2	Unassigned	Unassigned	Gp2	Unassigned
ASV_116	Bacteria	Proteobacteria	Alphaproteobacteria	Rhodospirillales	Reyranellaceae	Reyranella	Unassigned
ASV_120	Bacteria	Proteobacteria	Alphaproteobacteria	Rhodospirillales	Azospirillaceae	Skermanella	Unassigned
ASV_121	Bacteria	Proteobacteria	Betaproteobacteria	Burkholderiales	Unassigned	Unassigned	Unassigned
ASV_122	Bacteria	Acidobacteria	Acidobacteria_Gp3	Unassigned	Unassigned	Gp3	Unassigned
ASV_127	Bacteria	Proteobacteria	Betaproteobacteria	Rhodocyclales	Zoogloeaceae	Unassigned	Unassigned
ASV_138	Bacteria	Firmicutes	Clostridia	Clostridiales	Unassigned	Unassigned	Unassigned
ASV_151	Bacteria	Acidobacteria	Acidobacteria_Gp2	Unassigned	Unassigned	Gp2	Unassigned
ASV_152	Bacteria	Proteobacteria	Gammaproteobacteria	Gammaproteobacteria_incertae_sedis	Unassigned	Acidibacter	Unassigned
ASV_155	Bacteria	Acidobacteria	Acidobacteria_Gp1	Unassigned	Unassigned	Gp1	Unassigned
ASV_162	Bacteria	Proteobacteria	Alphaproteobacteria	Micropepsales	Micropepsaceae	Rhizomicrobium	Unassigned
ASV_178	Bacteria	Proteobacteria	Betaproteobacteria	Burkholderiales	Burkholderiaceae	Caballeronia	Unassigned
ASV_188	Bacteria	Proteobacteria	Alphaproteobacteria	Rhodospirillales	Azospirillaceae	Nitrospirillum	Unassigned
ASV_206	Bacteria	Acidobacteria	Acidobacteria_Gp3	Unassigned	Unassigned	Gp3	Unassigned
ASV_247	Bacteria	Acidobacteria	Acidobacteria_Gp3	Unassigned	Unassigned	Gp3	Unassigned

The shared strains from the rhizosphere soil of NP, SP, and DP*C. cathayensis* trees

OTU	Kingdom	Phylum	Class	Order	Family	Genus	Species
ASV_1	Bacteria	Proteobacteria	Gammaproteobacteria	Pseudomonadales	Pseudomonadaceae	Pseudomonas	Unassigned
ASV_2	Bacteria	Acidobacteria	Acidobacteria_Gp2	Unassigned	Unassigned	Gp2	Unassigned
ASV_4	Bacteria	Verrucomicrobia	Spartobacteria	Unassigned	Unassigned	Spartobacteria_genera_incertae_sedis	Unassigned
ASV_7	Bacteria	Gemmatimonadetes	Gemmatimonadetes	Gemmatimonadales	Gemmatimonadaceae	Gemmatimonas	Unassigned
ASV_8	Bacteria	Proteobacteria	Betaproteobacteria	Neisseriales	Chromobacteriaceae	Vogesella	Unassigned
ASV_9	Bacteria	Actinobacteria	Thermoleophilia	Gaiellales	Gaiellaceae	Gaiella	Unassigned
ASV_10	Bacteria	Proteobacteria	Alphaproteobacteria	Rhizobiales	Bradyrhizobiaceae	Bradyrhizobium	Unassigned
ASV_11	Bacteria	Acidobacteria	Acidobacteria_Gp1	Unassigned	Unassigned	Gp1	Unassigned
ASV_12	Archaea	Thaumarchaeota	Unassigned	Nitrososphaerales	Nitrososphaeraceae	Nitrososphaera	Unassigned
ASV_13	Bacteria	Firmicutes	Bacilli	Bacillales	Bacillaceae_1	Bacillus	Unassigned
ASV_14	Bacteria	Proteobacteria	Alphaproteobacteria	Micropepsales	Micropepsaceae	Micropepsis	Unassigned
ASV_15	Bacteria	Acidobacteria	Unassigned	Unassigned	Unassigned	Unassigned	Unassigned
ASV_16	Bacteria	Firmicutes	Bacilli	Bacillales	Unassigned	Unassigned	Unassigned
ASV_17	Bacteria	Proteobacteria	Betaproteobacteria	Neisseriales	Chromobacteriaceae	Formivibrio	Unassigned
ASV_18	Bacteria	Acidobacteria	Acidobacteria_Gp1	Unassigned	Unassigned	Gp1	Unassigned
ASV_19	Bacteria	Proteobacteria	Betaproteobacteria	Burkholderiales	Burkholderiaceae	Robbsia	Unassigned
ASV_23	Bacteria	Proteobacteria	Alphaproteobacteria	Rhizobiales	Bradyrhizobiaceae	Bradyrhizobium	Unassigned
ASV_24	Bacteria	Proteobacteria	Alphaproteobacteria	Rhizobiales	Bradyrhizobiaceae	Pseudolabrys	Unassigned
ASV_25	Bacteria	Acidobacteria	Acidobacteria_Gp2	Unassigned	Unassigned	Gp2	Unassigned
ASV_26	Bacteria	Proteobacteria	Alphaproteobacteria	Caulobacterales	Caulobacteraceae	Phenylobacterium	Unassigned
ASV_27	Bacteria	Proteobacteria	Gammaproteobacteria	Gammaproteobacteria_incertae_sedis	Unassigned	Acidibacter	Unassigned
ASV_29	Bacteria	Proteobacteria	Alphaproteobacteria	Micropepsales	Micropepsaceae	Micropepsis	Unassigned
ASV_30	Bacteria	Acidobacteria	Acidobacteria_Gp6	Unassigned	Unassigned	Gp6	Unassigned
ASV_31	Bacteria	Acidobacteria	Acidobacteria_Gp2	Unassigned	Unassigned	Gp2	Unassigned
ASV_32	Bacteria	Firmicutes	Bacilli	Bacillales	Unassigned	Unassigned	Unassigned
ASV_33	Bacteria	Verrucomicrobia	Spartobacteria	Unassigned	Unassigned	Spartobacteria_genera_incertae_sedis	Unassigned
ASV_34	Bacteria	Gemmatimonadetes	Gemmatimonadetes	Gemmatimonadales	Gemmatimonadaceae	Gemmatirosa	Unassigned
ASV_35	Bacteria	Proteobacteria	Alphaproteobacteria	Rhizobiales	Bradyrhizobiaceae	Bradyrhizobium	Unassigned
ASV_37	Bacteria	Actinobacteria	Thermoleophilia	Gaiellales	Gaiellaceae	Gaiella	Unassigned
ASV_38	Bacteria	Acidobacteria	Acidobacteria_Gp1	Unassigned	Unassigned	Gp1	Unassigned
ASV_40	Bacteria	Proteobacteria	Betaproteobacteria	Neisseriales	Neisseriaceae	Crenobacter	Unassigned
ASV_42	Bacteria	Proteobacteria	Betaproteobacteria	Burkholderiales	Oxalobacteraceae	Massilia	Unassigned
ASV_44	Bacteria	Firmicutes	Bacilli	Bacillales	Unassigned	Unassigned	Unassigned
ASV_48	Bacteria	Acidobacteria	Acidobacteria_Gp1	Unassigned	Unassigned	Gp1	Unassigned
ASV_49	Bacteria	Proteobacteria	Gammaproteobacteria	Gammaproteobacteria_incertae_sedis	Unassigned	Acidibacter	Unassigned
ASV_50	Bacteria	Acidobacteria	Acidobacteria_Gp1	Unassigned	Unassigned	Gp1	Unassigned
ASV_51	Bacteria	Actinobacteria	Thermoleophilia	Gaiellales	Gaiellaceae	Gaiella	Unassigned
ASV_52	Bacteria	Proteobacteria	Betaproteobacteria	Nitrosomonadales	Thiobacillaceae	Sulfuritortus	Unassigned
ASV_56	Bacteria	Proteobacteria	Alphaproteobacteria	Rhodospirillales	Reyranellaceae	Reyranella	Unassigned
ASV_57	Bacteria	Proteobacteria	Betaproteobacteria	Burkholderiales	Burkholderiaceae	Paraburkholderia	Unassigned
ASV_58	Bacteria	Acidobacteria	Acidobacteria_Gp2	Unassigned	Unassigned	Gp2	Unassigned
ASV_59	Bacteria	Acidobacteria	Acidobacteria_Gp1	Unassigned	Unassigned	Gp1	Unassigned
ASV_60	Bacteria	Proteobacteria	Alphaproteobacteria	Rhodospirillales	Azospirillaceae	Azospirillum	Unassigned
ASV_61	Bacteria	Acidobacteria	Acidobacteria_Gp2	Unassigned	Unassigned	Gp2	Unassigned
ASV_62	Bacteria	Actinobacteria	Actinobacteria	Streptosporangiales	Thermomonosporaceae	Actinoallomurus	Unassigned
ASV_66	Bacteria	Acidobacteria	Acidobacteria_Gp7	Unassigned	Unassigned	Gp7	Unassigned
ASV_68	Bacteria	Proteobacteria	Betaproteobacteria	Neisseriales	Chromobacteriaceae	Unassigned	Unassigned
ASV_71	Bacteria	Actinobacteria	Actinobacteria	Streptosporangiales	Thermomonosporaceae	Unassigned	Unassigned
ASV_73	Bacteria	Proteobacteria	Betaproteobacteria	Nitrosomonadales	Thiobacillaceae	Sulfuritortus	Unassigned
ASV_79	Bacteria	Proteobacteria	Alphaproteobacteria	Rhodospirillales	Unassigned	Unassigned	Unassigned
ASV_81	Bacteria	Proteobacteria	Alphaproteobacteria	Rhodospirillales	Azospirillaceae	Azospirillum	Unassigned
ASV_82	Bacteria	Acidobacteria	Acidobacteria_Gp1	Unassigned	Unassigned	Gp1	Unassigned
ASV_85	Bacteria	Proteobacteria	Alphaproteobacteria	Rhodospirillales	Unassigned	Unassigned	Unassigned
ASV_87	Bacteria	Proteobacteria	Gammaproteobacteria	Gammaproteobacteria_incertae_sedis	Unassigned	Acidibacter	Unassigned
ASV_88	Bacteria	Acidobacteria	Acidobacteria_Gp13	Unassigned	Unassigned	Gp13	Unassigned
ASV_89	Bacteria	Proteobacteria	Alphaproteobacteria	Rhizobiales	Bradyrhizobiaceae	Pseudolabrys	Unassigned
ASV_100	Bacteria	Proteobacteria	Alphaproteobacteria	Rhizobiales	Bradyrhizobiaceae	Pseudolabrys	Unassigned
ASV_102	Bacteria	Acidobacteria	Acidobacteria_Gp2	Unassigned	Unassigned	Gp2	Unassigned
ASV_104	Bacteria	Proteobacteria	Alphaproteobacteria	Sphingomonadales	Sphingomonadaceae	Sphingomonas	Unassigned
ASV_107	Bacteria	Nitrospirae	Nitrospira	Nitrospirales	Nitrospiraceae	Nitrospira	Unassigned
ASV_124	Bacteria	Proteobacteria	Deltaproteobacteria	Myxococcales	Labilitrichaceae	Labilithrix	Unassigned
ASV_125	Bacteria	Proteobacteria	Alphaproteobacteria	Rhizobiales	Bradyrhizobiaceae	Pseudorhodoplanes	Unassigned
ASV_127	Bacteria	Proteobacteria	Betaproteobacteria	Burkholderiales	Burkholderiaceae	Paraburkholderia	Unassigned
ASV_136	Bacteria	Proteobacteria	Betaproteobacteria	Burkholderiales	Burkholderiales_incertae_sedis	Thiobacter	Unassigned
ASV_138	Bacteria	Actinobacteria	Thermoleophilia	Gaiellales	Gaiellaceae	Gaiella	Unassigned
ASV_207	Bacteria	Proteobacteria	Alphaproteobacteria	Rhodospirillales	Azospirillaceae	Skermanella	Unassigned
ASV_2461	Bacteria	Proteobacteria	Deltaproteobacteria	Myxococcales	Myxococcaceae	Aggregicoccus	Unassigned

The shared strains from the rhizosphere soil of SP and DP*C. cathayensis* trees

OTU	Kingdom	Phylum	Class	Order	Family	Genus	Species
ASV_3	Bacteria	Firmicutes	Unassigned	Unassigned	Unassigned	Unassigned	Unassigned
ASV_20	Bacteria	Acidobacteria	Acidobacteria_Gp2	Unassigned	Unassigned	Gp2	Unassigned
ASV_28	Archaea	Thaumarchaeota	Unassigned	Nitrososphaerales	Nitrososphaeraceae	Nitrososphaera	Unassigned
ASV_36	Bacteria	Proteobacteria	Unassigned	Unassigned	Unassigned	Unassigned	Unassigned
ASV_41	Archaea	Thaumarchaeota	Unassigned	Nitrososphaerales	Nitrososphaeraceae	Nitrososphaera	Unassigned
ASV_53	Bacteria	Proteobacteria	Gammaproteobacteria	Chromatiales	Ectothiorhodospiraceae	Unassigned	Unassigned
ASV_54	Bacteria	Proteobacteria	Alphaproteobacteria	Rhodospirillales	Azospirillaceae	Nitrospirillum	Unassigned
ASV_64	Bacteria	Proteobacteria	Betaproteobacteria	Burkholderiales	Burkholderiaceae	Robbsia	Unassigned
ASV_67	Bacteria	Actinobacteria	Actinobacteria	Unassigned	Unassigned	Unassigned	Unassigned
ASV_72	Bacteria	Acidobacteria	Acidobacteria_Gp2	Unassigned	Unassigned	Gp2	Unassigned
ASV_74	Archaea	Thaumarchaeota	Unassigned	Nitrosopumilales	Nitrosopumilaceae	Nitrosopumilus	Unassigned
ASV_76	Bacteria	Acidobacteria	Acidobacteria_Gp2	Unassigned	Unassigned	Gp2	Unassigned
ASV_95	Bacteria	Proteobacteria	Betaproteobacteria	Burkholderiales	Burkholderiaceae	Trinickia	Unassigned
ASV_98	Bacteria	Verrucomicrobia	Spartobacteria	Unassigned	Unassigned	Spartobacteria_genera_incertae_sedis	Unassigned
ASV_111	Bacteria	Verrucomicrobia	Spartobacteria	Unassigned	Unassigned	Spartobacteria_genera_incertae_sedis	Unassigned
ASV_116	Bacteria	Proteobacteria	Gammaproteobacteria	Unassigned	Unassigned	Unassigned	Unassigned
ASV_123	Bacteria	Acidobacteria	Acidobacteria_Gp1	Unassigned	Unassigned	Gp1	Unassigned
ASV_128	Bacteria	Actinobacteria	Actinobacteria	Unassigned	Unassigned	Unassigned	Unassigned
ASV_159	Bacteria	Proteobacteria	Alphaproteobacteria	Rhizobiales	Bradyrhizobiaceae	Pseudolabrys	Unassigned
ASV_163	Bacteria	Acidobacteria	Acidobacteria_Gp1	Unassigned	Unassigned	Gp1	Unassigned
ASV_171	Bacteria	Acidobacteria	Acidobacteria_Gp2	Unassigned	Unassigned	Gp2	Unassigned
ASV_172	Bacteria	Acidobacteria	Acidobacteria_Gp1	Unassigned	Unassigned	Gp1	Unassigned
ASV_174	Bacteria	Actinobacteria	Thermoleophilia	Solirubrobacterales	Conexibacteraceae	Conexibacter	Unassigned
ASV_213	Bacteria	Acidobacteria	Acidobacteria_Gp3	Unassigned	Unassigned	Gp3	Unassigned

The shared strains from the bulk soil of NP, SP, and DP *C. cathayensis* trees

OTU	Kingdom	Phylum	Class	Order	Family	Genus	Species
ASV_1	Bacteria	Acidobacteria	Acidobacteria_Gp2	Unassigned	Unassigned	Gp2	Unassigned
ASV_2	Bacteria	Proteobacteria	Gammaproteobacteria	Pseudomonadales	Pseudomonadaceae	Pseudomonas	Unassigned
ASV_3	Bacteria	Acidobacteria	Acidobacteria_Gp1	Unassigned	Unassigned	Gp1	Unassigned
ASV_4	Bacteria	Proteobacteria	Gammaproteobacteria	Gammaproteobacteria_incertae_sedis	Unassigned	Acidibacter	Unassigned
ASV_5	Bacteria	Verrucomicrobia	Spartobacteria	Unassigned	Unassigned	Spartobacteria_genera_incertae_sedis	Unassigned
ASV_6	Bacteria	Proteobacteria	Betaproteobacteria	Neisseriales	Chromobacteriaceae	Formivibrio	Unassigned
ASV_7	Bacteria	Proteobacteria	Alphaproteobacteria	Rhizobiales	Bradyrhizobiaceae	Bradyrhizobium	Unassigned
ASV_8	Bacteria	Acidobacteria	Acidobacteria_Gp1	Unassigned	Unassigned	Gp1	Unassigned
ASV_9	Bacteria	Acidobacteria	Acidobacteria_Gp1	Unassigned	Unassigned	Gp1	Unassigned
ASV_10	Archaea	Thaumarchaeota	Unassigned	Nitrososphaerales	Nitrososphaeraceae	Nitrososphaera	Unassigned
ASV_11	Bacteria	Proteobacteria	Alphaproteobacteria	Micropepsales	Micropepsaceae	Micropepsis	Unassigned
ASV_13	Bacteria	Actinobacteria	Thermoleophilia	Gaiellales	Gaiellaceae	Gaiella	Unassigned
ASV_14	Bacteria	Acidobacteria	Acidobacteria_Gp1	Unassigned	Unassigned	Gp1	Unassigned
ASV_15	Bacteria	Acidobacteria	Acidobacteria_Gp2	Unassigned	Unassigned	Gp2	Unassigned
ASV_16	Bacteria	Acidobacteria	Acidobacteria_Gp2	Unassigned	Unassigned	Gp2	Unassigned
ASV_17	Bacteria	Firmicutes	Bacilli	Bacillales	Bacillaceae_1	Bacillus	Unassigned
ASV_19	Bacteria	Proteobacteria	Betaproteobacteria	Burkholderiales	Burkholderiaceae	Robbsia	Unassigned
ASV_20	Bacteria	Proteobacteria	Alphaproteobacteria	Rhodospirillales	Rhodospirillaceae	Magnetospirillum	Unassigned
ASV_21	Bacteria	Armatimonadetes	Unassigned	Unassigned	Unassigned	Unassigned	Unassigned
ASV_22	Bacteria	Proteobacteria	Unassigned	Unassigned	Unassigned	Unassigned	Unassigned
ASV_23	Bacteria	Armatimonadetes	Unassigned	Unassigned	Unassigned	Armatimonadetes_gp7	Unassigned
ASV_24	Bacteria	Proteobacteria	Betaproteobacteria	Neisseriales	Chromobacteriaceae	Formivibrio	Unassigned
ASV_25	Bacteria	Proteobacteria	Betaproteobacteria	Nitrosomonadales	Sterolibacteriaceae	Denitratisoma	Unassigned
ASV_26	Bacteria	Proteobacteria	Alphaproteobacteria	Rhizobiales	Bradyrhizobiaceae	Bradyrhizobium	Unassigned
ASV_27	Bacteria	Proteobacteria	Alphaproteobacteria	Micropepsales	Micropepsaceae	Rhizomicrobium	Unassigned
ASV_28	Bacteria	Proteobacteria	Gammaproteobacteria	Gammaproteobacteria_incertae_sedis	Unassigned	Acidibacter	Unassigned
ASV_29	Bacteria	Acidobacteria	Acidobacteria_Gp6	Unassigned	Unassigned	Gp6	Unassigned
ASV_30	Bacteria	Firmicutes	Clostridia	Halanaerobiales	Halanaerobiaceae	Halothermothrix	Unassigned
ASV_31	Bacteria	Proteobacteria	Alphaproteobacteria	Rhodospirillales	Reyranellaceae	Reyranella	Unassigned
ASV_32	Bacteria	Acidobacteria	Acidobacteria_Gp2	Unassigned	Unassigned	Gp2	Unassigned
ASV_33	Bacteria	Acidobacteria	Acidobacteria_Gp1	Unassigned	Unassigned	Gp1	Unassigned
ASV_34	Bacteria	Firmicutes	Bacilli	Bacillales	Bacillaceae_1	Neobacillus	Unassigned
ASV_36	Bacteria	Proteobacteria	Betaproteobacteria	Burkholderiales	Burkholderiaceae	Paraburkholderia	Unassigned
ASV_37	Bacteria	Acidobacteria	Acidobacteria_Gp1	Unassigned	Unassigned	Gp1	Unassigned
ASV_39	Bacteria	Proteobacteria	Betaproteobacteria	Nitrosomonadales	Thiobacillaceae	Sulfuritortus	Unassigned
ASV_40	Bacteria	Acidobacteria	Acidobacteria_Gp2	Unassigned	Unassigned	Gp2	Unassigned
ASV_41	Bacteria	Gemmatimonadetes	Gemmatimonadetes	Gemmatimonadales	Gemmatimonadaceae	Gemmatimonas	Unassigned
ASV_42	Bacteria	Acidobacteria	Acidobacteria_Gp1	Unassigned	Unassigned	Gp1	Unassigned
ASV_43	Bacteria	Firmicutes	Bacilli	Bacillales	Bacillales_incertae_sedis	Caldalkalibacillus	Unassigned
ASV_46	Bacteria	Acidobacteria	Acidobacteria_Gp2	Unassigned	Unassigned	Gp2	Unassigned
ASV_47	Bacteria	Acidobacteria	Acidobacteria_Gp1	Unassigned	Unassigned	Unassigned	Unassigned
ASV_48	Bacteria	Verrucomicrobia	Spartobacteria	Unassigned	Unassigned	Spartobacteria_genera_incertae_sedis	Unassigned
ASV_50	Bacteria	Gemmatimonadetes	Gemmatimonadetes	Gemmatimonadales	Gemmatimonadaceae	Gemmatimonas	Unassigned
ASV_51	Bacteria	Proteobacteria	Alphaproteobacteria	Rhizobiales	Bradyrhizobiaceae	Pseudolabrys	Unassigned
ASV_52	Bacteria	Acidobacteria	Acidobacteria_Gp2	Unassigned	Unassigned	Gp2	Unassigned
ASV_53	Bacteria	Proteobacteria	Alphaproteobacteria	Rhodospirillales	Azospirillaceae	Skermanella	Unassigned
ASV_54	Bacteria	Proteobacteria	Alphaproteobacteria	Rhizobiales	Bradyrhizobiaceae	Bradyrhizobium	Unassigned
ASV_55	Bacteria	Proteobacteria	Gammaproteobacteria	Gammaproteobacteria_incertae_sedis	Unassigned	Acidibacter	Unassigned
ASV_56	Bacteria	Acidobacteria	Acidobacteria_Gp7	Unassigned	Unassigned	Gp7	Unassigned
ASV_58	Bacteria	Proteobacteria	Betaproteobacteria	Nitrosomonadales	Thiobacillaceae	Sulfuritortus	Unassigned
ASV_60	Bacteria	Acidobacteria	Acidobacteria_Gp2	Unassigned	Unassigned	Gp2	Unassigned
ASV_61	Bacteria	Proteobacteria	Betaproteobacteria	Burkholderiales	Burkholderiaceae	Paraburkholderia	Unassigned
ASV_62	Bacteria	Acidobacteria	Acidobacteria_Gp2	Unassigned	Unassigned	Gp2	Unassigned
ASV_64	Bacteria	Proteobacteria	Betaproteobacteria	Neisseriales	Chromobacteriaceae	Vogesella	Unassigned
ASV_65	Bacteria	Proteobacteria	Alphaproteobacteria	Sphingomonadales	Sphingomonadaceae	Sphingomonas	Unassigned
ASV_66	Bacteria	Nitrospirae	Nitrospira	Nitrospirales	Nitrospiraceae	Nitrospira	Unassigned
ASV_67	Bacteria	Gemmatimonadetes	Gemmatimonadetes	Gemmatimonadales	Gemmatimonadaceae	Gemmatimonas	Unassigned
ASV_69	Bacteria	Proteobacteria	Betaproteobacteria	Rhodocyclales	Rhodocyclaceae	Propionivibrio	Unassigned
ASV_70	Bacteria	Actinobacteria	Actinobacteria	Streptosporangiales	Thermomonosporaceae	Actinoallomurus	Unassigned
ASV_75	Bacteria	Acidobacteria	Acidobacteria_Gp3	Unassigned	Unassigned	Gp3	Unassigned
ASV_76	Bacteria	Proteobacteria	Alphaproteobacteria	Rhodospirillales	Azospirillaceae	Skermanella	Unassigned
ASV_77	Bacteria	Proteobacteria	Alphaproteobacteria	Rhodospirillales	Azospirillaceae	Unassigned	Unassigned
ASV_78	Bacteria	Acidobacteria	Acidobacteria_Gp2	Unassigned	Unassigned	Gp2	Unassigned
ASV_80	Bacteria	Acidobacteria	Acidobacteria_Gp3	Unassigned	Unassigned	Gp3	Unassigned
ASV_83	Bacteria	Actinobacteria	Actinobacteria	Streptosporangiales	Thermomonosporaceae	Actinomadura	Unassigned
ASV_85	Bacteria	Proteobacteria	Alphaproteobacteria	Rhizobiales	Bradyrhizobiaceae	Rhodoplanes	Unassigned
ASV_87	Bacteria	Verrucomicrobia	Subdivision3	Unassigned	Unassigned	Subdivision3_genera_incertae_sedis	Unassigned
ASV_94	Bacteria	Acidobacteria	Acidobacteria_Gp1	Unassigned	Unassigned	Gp1	Unassigned
ASV_95	Bacteria	Proteobacteria	Betaproteobacteria	Burkholderiales	Oxalobacteraceae	Massilia	Unassigned
ASV_98	Bacteria	Proteobacteria	Gammaproteobacteria	Gammaproteobacteria_incertae_sedis	Unassigned	Acidibacter	Unassigned
ASV_99	Bacteria	Proteobacteria	Alphaproteobacteria	Rhodospirillales	Rhodospirillaceae	Ferruginivarius	Unassigned
ASV_103	Bacteria	Proteobacteria	Alphaproteobacteria	Rhodospirillales	Acetobacteraceae	Unassigned	Unassigned
ASV_106	Bacteria	Actinobacteria	Thermoleophilia	Gaiellales	Gaiellaceae	Gaiella	Unassigned
ASV_108	Bacteria	Proteobacteria	Betaproteobacteria	Nitrosomonadales	Thiobacillaceae	Sulfuritortus	Unassigned
ASV_112	Bacteria	Proteobacteria	Alphaproteobacteria	Rhizobiales	Bradyrhizobiaceae	Pseudolabrys	Unassigned
ASV_120	Bacteria	Acidobacteria	Acidobacteria_Gp1	Unassigned	Unassigned	Gp1	Unassigned
ASV_123	Bacteria	Acidobacteria	Acidobacteria_Gp1	Unassigned	Unassigned	Gp1	Unassigned
ASV_125	Bacteria	Proteobacteria	Gammaproteobacteria	Pseudomonadales	Moraxellaceae	Perlucidibaca	Unassigned
ASV_126	Bacteria	Proteobacteria	Betaproteobacteria	Burkholderiales	Burkholderiales_incertae_s	Thiobacter	Unassigned
ASV_134	Bacteria	Proteobacteria	Alphaproteobacteria	Rhizobiales	Bradyrhizobiaceae	Pseudolabrys	Unassigned
ASV_167	Bacteria	Proteobacteria	Deltaproteobacteria	Myxococcales	Labilitrichaceae	Labilithrix	Unassigned
ASV_204	Bacteria	Acidobacteria	Unassigned	Unassigned	Unassigned	Unassigned	Unassigned
ASV_469	Bacteria	Proteobacteria	Alphaproteobacteria	Rhodospirillales	Azospirillaceae	Skermanella	Unassigned
ASV_2090	Bacteria	Proteobacteria	Deltaproteobacteria	Myxococcales	Myxococcaceae	Aggregicoccus	Unassigned

The shared strains from the bulk soil of SP and DP *C. cathayensis* trees

OTU	Kingdom	Phylum	Class	Order	Family	Genus	Species
ASV_18	Bacteria	Acidobacteria	Acidobacteria_Gp1	Unassigned	Unassigned	Gp1	Unassigned
ASV_35	Bacteria	Proteobacteria	Alphaproteobacteria	Rhodospirillales	Acetobacteraceae	Acidisoma	Unassigned
ASV_49	Bacteria	Proteobacteria	Betaproteobacteria	Nitrosomonadales	Sterolibacteriaceae	Sterolibacterium	Unassigned
ASV_86	Bacteria	Acidobacteria	Acidobacteria_Gp1	Unassigned	Unassigned	Gp1	Unassigned
ASV_88	Bacteria	Acidobacteria	Acidobacteria_Gp1	Unassigned	Unassigned	Gp1	Unassigned
ASV_89	Bacteria	Proteobacteria	Gammaproteobacteria	Gammaproteobacteria_incertae_sedis	Unassigned	Acidibacter	Unassigned
ASV_91	Bacteria	Unassigned	Unassigned	Unassigned	Unassigned	Unassigned	Unassigned
ASV_92	Bacteria	Acidobacteria	Acidobacteria_Gp10	Unassigned	Unassigned	Gp10	Unassigned
ASV_102	Bacteria	Proteobacteria	Gammaproteobacteria	Vibrionales	Vibrionaceae	Vibrio	Unassigned
ASV_104	Bacteria	Acidobacteria	Acidobacteria_Gp1	Unassigned	Unassigned	Gp1	Unassigned
ASV_109	Bacteria	Proteobacteria	Betaproteobacteria	Burkholderiales	Alcaligenaceae	Basilea	Unassigned
ASV_113	Bacteria	Acidobacteria	Acidobacteria_Gp1	Unassigned	Unassigned	Gp1	Unassigned
ASV_116	Bacteria	Acidobacteria	Acidobacteria_Gp1	Unassigned	Unassigned	Gp1	Unassigned
ASV_118	Bacteria	Proteobacteria	Gammaproteobacteria	Gammaproteobacteria_incertae_sedis	Unassigned	Acidibacter	Unassigned
ASV_119	Bacteria	Proteobacteria	Alphaproteobacteria	Rhodospirillales	Acetobacteraceae	Acidisphaera	Unassigned
ASV_121	Bacteria	Acidobacteria	Acidobacteria_Gp1	Unassigned	Unassigned	Gp1	Unassigned
ASV_138	Bacteria	Acidobacteria	Acidobacteria_Gp1	Unassigned	Unassigned	Gp1	Unassigned
ASV_141	Bacteria	Acidobacteria	Acidobacteria_Gp2	Unassigned	Unassigned	Gp2	Unassigned
ASV_143	Bacteria	Proteobacteria	Alphaproteobacteria	Micropepsales	Micropepsaceae	Rhizomicrobium	Unassigned
ASV_156	Bacteria	Proteobacteria	Betaproteobacteria	Neisseriales	Chromobacteriaceae	Vogesella	Unassigned
ASV_157	Bacteria	Proteobacteria	Alphaproteobacteria	Caulobacterales	Caulobacteraceae	Phenylobacterium	Unassigned
ASV_168	Bacteria	Proteobacteria	Gammaproteobacteria	Cellvibrionales	Cellvibrionaceae	Unassigned	Unassigned
ASV_190	Bacteria	Actinobacteria	Thermoleophilia	Gaiellales	Gaiellaceae	Gaiella	Unassigned
ASV_197	Bacteria	Actinobacteria	Actinobacteria	Streptosporangiales	Thermomonosporaceae	Actinoallomurus	Unassigned
